# The Emerging Role of Senotherapy in Cancer: A Comprehensive Review

**DOI:** 10.3390/clinpract13040076

**Published:** 2023-07-22

**Authors:** Sarubala Malayaperumal, Francesco Marotta, Makalakshmi Murali Kumar, Indumathi Somasundaram, Antonio Ayala, Mario Munoz Pinto, Antara Banerjee, Surajit Pathak

**Affiliations:** 1 Faculty of Allied Health Sciences, Chettinad Academy of Research and Education (CARE), Kelambakkam, Chennai 603103, India; sarubala18@gmail.com (S.M.); makalakshmimk@gmail.com (M.M.K.); antara.banerjee27@gmail.com (A.B.); 2 ReGenera R&D International for Aging Intervention, 20154 Milan, Italy; 3 Department of Biotech, KIT College of Engineering, Kolhapur, Maharashtra 416013, India; drindumathisomasundaram@gmail.com; 4 Department of Biochemistry and Molecular Biology, University of Seville, 41012 Seville, Spain; aayala@us.es (A.A.); mariomunoz@us.es (M.M.P.)

**Keywords:** SASP, cancer, senolytes, senolytic drugs, cellular senescence

## Abstract

Senotherapy, a promising therapeutic strategy, has drawn a lot attention recently due to its potential for combating cancer. Senotherapy refers to the targeting of senescent cells to restore tissue homeostasis and mitigate the deleterious effects associated with senescence. Senolytic drugs represent a promising avenue in cancer treatment, with the potential to target and modulate senescent cells to improve patient outcomes. The review highlights the intricate interplay between the senescence-associated secretory phenotype (SASP) and the tumor microenvironment, emphasizing the role of senescent cells in promoting chronic inflammation, immune evasion, and tumor-cell proliferation. It then explores the potential of senotherapy as a novel strategy for cancer therapy. This review addresses the emerging evidence on the combination of senotherapy with conventional cancer treatments, such as chemotherapy and immunotherapy.

## 1. Introduction

Cellular senescence is defined as a state in which proliferating cells stop dividing with sustained viability, and show increased metabolic activity due to DNA damage and resistance to growth-promoting stimuli and apoptosis [1]. Leonard Hayflick and Moorehead, in 1961, first reported the process of cellular senescence. Signals that can subsidize cells entering the senescent phase comprise signals from cellular damage or cancer progression [1,2]. It is a natural anti-tumor mechanism that perpetually halts cells at risk for malignant transformation [3]. However, senescent cells can procure a senescence-associated secretory phenotype (SASP) or senescence-messaging secretome (SMS), which are also called deleterious senescent cells. Secretory phenotype cells are not limited to the cell-cycle arrest, but are metabolically active cells that have changed protein expression and secretion [4]. The acquisition of SASP leads senescent cells to turn into pro-inflammatory cells, which can promote tumorigenesis [3]. Pro-apoptosis, inflammation, and insulin resistance are some of the senescence-associated secretory phenotypes [5]. Studies have reported that the presence of SASP is biologically associated with numerous cytokines such as TNF-alpha, growth factors that support cancer progression, matrix metalloproteinases (MMPs), such as MMP-3 and MMP-9, secreted proteins, chemokines that activate immune cells, lipids that contribute to inflammation and tissue damage, etc. [6]. Regulatory pathways such as the DNA damage response (DDR), unfolded protein response (UPR), cell cycle regulatory and apoptotic pathways that control cellular proliferation, apoptosis, and DNA damage are responsible for the stimulation of senescence and many of these pathways are involved in cancer development and progression [7,8].

Senolytic drugs can specifically aim at and clear the senescent cells and thus invigorate the tissues [9]. Navitoclax or ABT-263, quercetin, and fisetin were the first discovered senolytic drugs [1,9] and many other compounds such as danazol, nicotinamide, and riboside have been proven to have senolytic properties [10,11,12]. Senolytic drugs may have a part in abolishing this fibrotic response; however, to date there have been no proper data from human trials to validate this [7]. The accumulation of senescent cells (SNCs) over time can worsen the toxic side effects of treatments, increase treatment resistance, and lead to a poor prognosis [11]. Additionally, tumor cells that become senescent can develop stemness and repopulate the tumor, causing cancer recurrence. Therefore, senotherapies that eliminate SNCs may be a new strategy for antitumor therapy [12]. This review discusses the negative impact of SNCs on tumor development and how senescent tumor cells escape senescence. It also explores the link between senescence and polyploidy and highlights the potential of senotherapies as a promising adjuvant treatment for cancer. This approach is expected to provide new insights into antitumor drug development based on cellular senescence.

## 2. SASP (Senescence-Associated Secretory Phenotype)

A senescent cell which is metabolically active and has undergone extensive changes in protein expression and secretion leads to the development of SASP. It is also called a senescence-messaging secretome [4,13]. An extensive range of proteins, such as inflammatory cytokines, growth factors, cyclooxygenase, chemokines, and metalloproteinase, are secreted by these cells [3,14]. SASP factors released by senescent cells affect the neighboring non-senescent cells and the ECM (extracellular matrix), thereby eliciting inflammation, fibrosis, and the programmed cell death of the healthy cells, whereas senescent cells themselves become resistant to apoptosis, leading to the generation of more SASP factors [15].

**Figure 1:**
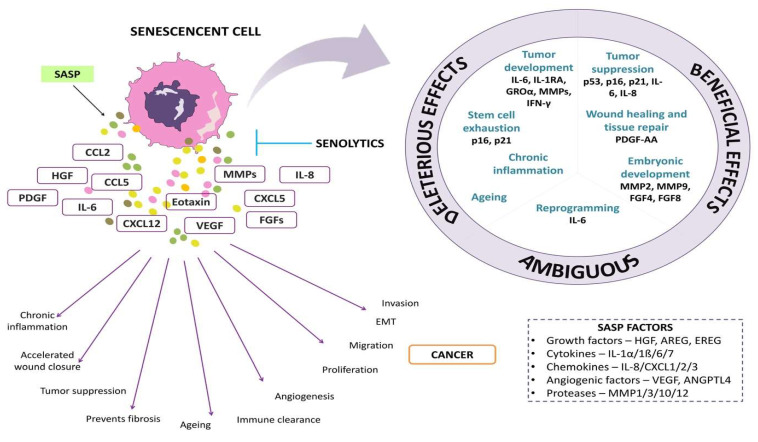
The representative image depicts senescence-associated secretory factors and their effects on cells.

The amount of SASP factor produced by a senescent cell varies according to the type and degree of the senescent stimulus. In a few cases, the generation of SASP may have a beneficial effect. For example, SASP includes growth factors such as vascular endothelial growth factor (VEGF), platelet-derived growth factor (PDGF), and insulin-like growth factor (IGF-1) which act in a paracrine manner on neighboring healthy cells, thus stimulating tissue repair and regeneration [16]. Secreted chemokines recruit immune cells to the site and clear the senescent cells, in a phenomenon called senescence surveillance. This mechanism is suggested to function to deplete pre-cancerous cells [17]. Figure 1 shows the overview of SASP factors and their effects on cells.

In the context of tumors, cancer cells tend to utilize these growth factors and also induce pro-tumorigenic effects on senescent cells [18]. Additionally, SASP was also shown to induce secretion factors that induce angiogenesis and epithelial–mesenchymal transition in tumor cells [18]. Schafer et al. found that compared to senescent epithelial cells and myoblasts, senescent endothelium cells, pre-adipocytes, and fibroblasts release a more substantial amount of SASP proteins, resulting in a more robust SASP [19]. The levels of SASP components in human plasma can be accurately measured, and their abundance increases significantly with age. According to Schafer et al. (2020), SASP components may be accurately measured in human plasma, and their circulating abundance dramatically rises with age. Their findings provide credence to the idea that circulating SASP factors are valuable potential biomarkers of biological age that may be used to assess risk for unfavorable health outcomes in an unbiased way independent of disease. This idea may have broad implications for clinical practice and clinical research [19]. In Wiley et al.’s [20] research, oxylipins were identified as a lipid-based component of the SASP, and their secretion is predicted to have significant physiological effects, in addition to the cell-autonomous action of dihomo15d PGJ2. The SASP and senescence are both dynamic features that change over time [20].

Senescent cells contribute to a senescence-associated secretory phenotype (SASP) that promotes disease progression by impairing tissue regeneration and fostering an inflammatory environment. It is still unknown whether the ECM changes are also associated with ageing and fibrotic disease because the senescent phenotype is linked to these conditions. ECM breakdown products that frequently behave as damage-associated molecular patterns (DAMPs) which enable pattern-recognizing receptors (PRRs) on cells of the innate immune response are released during remodeling and injury. It is intriguing to point out that numerous chronic fibrotic diseases have been found to have significantly higher levels of a variety of these ECM DAMPs [21,22]. Nuclear factor b (NF-B)-mediated pro-inflammatory cytokine release is induced by DAMP-activated PRRs, and this release profile is comparable to that of the SASP. Additionally, in fibroblasts, NF-B is a well-known master regulator of the SASP, which raises the possibility that DAMPs play a role in the regulation of cellular senescence [23]. The SASP of senescent cells in fibrosis is made up of pro-inflammatory cytokines, which are activated by the binding of ECM DAMPs to TLR2/4. Cell-cycle arrest is induced and apoptosis resistance is increased when ECM proteins bind to their corresponding receptors (integrins or RTK). Additionally, the senescent phenotype is reinforced by the upregulation of profibrotic/pro-inflammatory cytokines in an autocrine-dependent manner while also spreading senescence to nearby cells as part of a feedback loop [23]. The proliferation, migration, and contraction of fibroblasts are all influenced more by the ECM’s stiffness than other factors. Additionally, a stiffer ECM increases latent transforming growth factor (TGF) activation, which promotes fibrosis and has been associated with cellular senescence [24].

## 3. Senolytics

**Figure 2:**
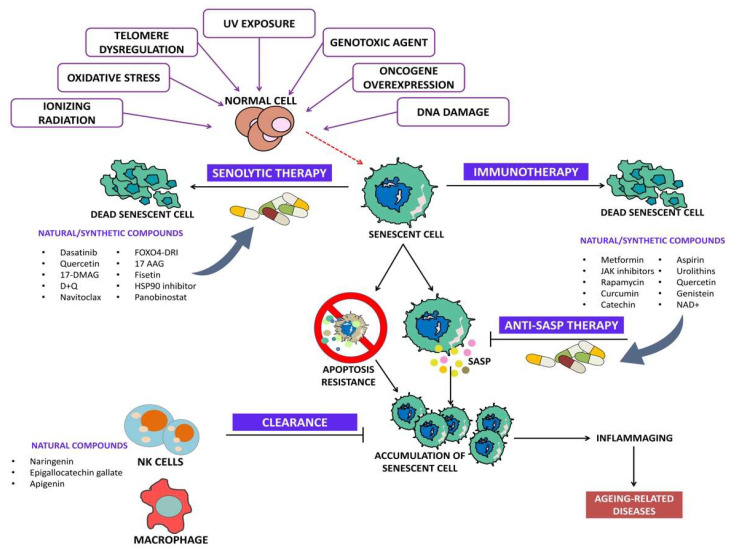
Overview of senolytic therapy.

Senolytics are drugs/agents that specifically induce programmed cell death in senescent cells. Senolytics can be categorized into (i) BCL family inhibitors, (ii) PI3K/AKT inhibitors, and (iii) FOXO regulators [25]. Figure 2 depicts the overview of senolytic therapy.

### 3.1. BCL Family Inhibitors

BCL family is composed of pro-survival and pro-apoptotic proteins such as BCL-XL and BCL-2. As BCL family proteins help in pro-survival, targeting them specifically aids to clear the senescent cells. Some of the BCL family inhibitors include navitoclax or ABT263, A1331852, A1155463, and ABT737 [25].

#### 3.1.1. Navitoclax

Navitoclax is a senolytic drug that targets the BCL pathway by binding to BCL-W, BCL-2, and BCL-XL. This drug can effectively decrease the survival of senescent lung fibroblast and umbilical vein epithelial cells, and it can be taken orally [26]. Studies have proved that sometimes chemotherapy causes senescence in a few cells and the administration of navitoclax as an adjuvant therapy has induced the senescent cells to undergo programmed cell death [27]. Adjuvant therapy with navitoclax not only promotes senescent cell apoptosis but also reduces the chances of recurrence and metastasis [28,29]. Though there is widespread evidence of this drug in clearing senescent cells, it is also reported to have severe side effects such as thrombocytopenia and neutropenia [10,30].

#### 3.1.2. Other BCL Inhibitors

ABT737, A1331852, and A1155463 are inhibitors of the BCL family of proteins. ABT737 is a precursor to navitoclax and acts as a BH3-mimetic, preventing the interaction between anti-apoptotic and pro-apoptotic proteins, leading to apoptosis in senescent cells. However, ABT737 has limited application due to its poor solubility and bioavailability. On the other hand, A1331852 and A1155463 have been identified as more effective candidates due to their specificity and lower toxicity [31].

##### Catechins

It is a polyphenolic compound found in green tea. Epigallocatechin gallate and epigallocatechin are the most abundant catechins found in green tea [32]. Epigallocatechin gallate controls incidences of inflammatory disorders and senescence. Further, catechins are strong antioxidants and free-radical scavengers owning a potential part in the management of various disorders [33]. A study reported that epigallocatechin gallate could inhibit SASP and protect cells against DNA damage and ROS by downregulating the PI3K/AKT pathway [34].

##### Panobinostat

Panobinostat is a type of histone deacetylase inhibitor that has properties that can fight against tumors, as well as remove senescent cells that accumulate during chemotherapy. Samaraweera et al. found that panobinostat can effectively eliminate senescent cells, possibly by inhibiting BCL-xL [35].

### 3.2. PI3K/AKT Inhibitors

Studies have reported that phosphoinositide 3-kinase (PI3K) protects the cells against programmed cell death and is also responsible for the pro-survival of senescent cells. Activation of PI3k can phosphorylate and inactivate Bad and caspase-9, which causes cell survival [36]. 

#### 3.2.1. Dasatinib + Quercetin

Dasatinib is a type of tyrosine kinase inhibitor that inhibits the proliferation, migration, and invasion of cells, and can also induce cancer-cell apoptosis. Quercetin is a natural flavonoid that can inhibit the activity of mTOR and PI3K. These two drugs were the first senolytic agents discovered based on a hypothesis-driven mechanism, and have been found to be beneficial in many diseases [9,37]. Along with quercetin and kaempferol, myricetin is a type of flavonoid that has garnered particular attention for its properties that include anti-photoaging, anti-cancer, and anti-platelet aggregation [38].

#### 3.2.2. Fisetin

This is a group of natural flavonoids found widely in a variety of fruits and vegetables such as persimmons, apples, onions, and strawberries, and they are well-known for their strong anti-oxidant property. Intake of a fisetin-rich diet has been proven to reduce vascular disease and decrease the risk of coronary heart disease [39,40]. Fisetin was proven to reduce the secretion of pro-inflammatory eicosanoids by inhibiting lipoxygenase activity, thus protecting the brain from neurological diseases [41]. Fisetin interferes with PI3K/AKT pathway and reduces the senescent cells, oxidative stress, and inflammation, thus reducing the risk of age-related disorders. Fisetin is available in the market for human consumption in low doses to enhance brain health [42,43].

### 3.3. FOXO4-DRI

FOXOs are a transcriptional factor that play a crucial role in cell survival, growth, and managing oxidative stress. They are involved in controlling various cellular processes such as DNA repair and apoptosis, and interact with the p53 gene. Findings have revealed that FOXO can interact with p53 and inhibit p53-mediated programmed cell death, thus maintaining the viability of senescent cells. To disrupt this interaction, a FOXO-DRI peptide was designed that has greater affinity to the p53 domain, breaking their interaction and inducing apoptosis in senescent cells [44].

### 3.4. Other Senolytics

Novel senolytic drugs with reduced cytotoxicity are needed, as existing senolytic drugs are highly toxic. To discover new agents that can safely target senescent cells, researchers have used a PDMA-based pharmacological library consisting mainly of natural compounds. This research led to the discovery of Procyanidin C1 (PCC1), a natural phytochemical agent with broad-spectrum senolytic activity. PCC1 also acts as a senomorphic agent, reducing SASP expression when used at low doses. PCC1 shares similar properties with grape seed extract, which can also have both senomorphic and senolytic effects. Natural procyanidins, like synthetic ones, possess anti-inflammatory, anti-arthritic, anti-allergic, and anticancer properties, and can scavenge oxygen free radicals and reduce radiation-induced peroxidation activity [45].

### 3.5. Phyto-Marine Senolytics

**Figure 3:**
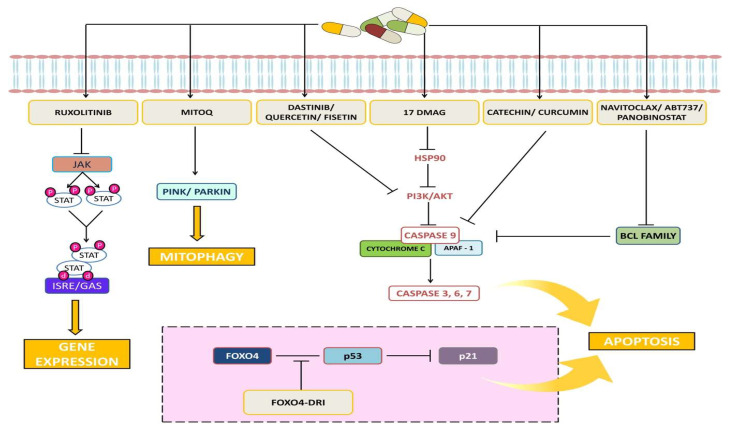
Representative image shows the targeted signaling pathways by senolytes.

Phyto-marine products refer to a newer combination of proteins obtained from plant root extract and marine fishes. For instance, Rhodiola Rosea is a perennial herb found in China, Russia, and Eastern Europe. It has a large root that is commonly known as “golden root” or “roseroot” due to its various pharmacological activities, including a rich variety of polyphenols, flavones, and esters of caffeic acids [46]. These properties contribute to its antioxidant, anti-inflammatory, antibacterial, cardio-protective, neuroprotective, longevity-improvement, and anticancer effects, making it a traditional medicinal herb. Rhodiola Rosea contains several bioactive compounds, including salidroside and tyrosal [46]. In our previous study, it was reported that the specific bioactive fractions from Rhodiola and lipoprotein isolated from Trachurus-species fish (R-L) (ReGenera International, Milan, Italy) have proved to enhance normal cell proliferation rate and also regulate vital genes [47]. Figure 3 shows the targeted signaling pathways by senolytes.

## 4. Senotherapy and Tumor Microenvironment (TME)

The TME has an essential role in the progression of cancer by contributing to therapeutic resistance, which is facilitated by intercellular communication. Various factors are responsible for activating survival pathways, immune escape, and the remodeling of the extracellular matrix, all of which induce resistance to cancer therapy [48]. In addition, cells in the TME often undergo therapy-induced senescence (TIS) as an unintended consequence of anticancer treatments. This can have significant impacts on the progression of the disease by promoting resistance and fueling the growth of the remaining cancer cells, leading to tumor relapse and distant metastasis [49]. SASP can be a key driver of the pathogenesis which facilitates communication between senescent surroundings and various immune-cell populations [50]. Senescent splenocyte stimulation in allogeneic mixed lymphocyte reactions leads to abnormalities in the proliferation of CD3+ T cells, which is also due to secreted SASP factors from the senescent splenic environment [51]. These interactions suggest that senescent stroma has the potential to destroy T cells, that is critical for tumor rejection [52]. Typically, inducing senescence in cancer cells could prevent malignancy. However, inducing senescence in genetically normal host cells within the TME frequently has the contradictory effect. The concept of immunosenescence, which refers to the senescence of immune cells, particularly T cell populations, is still being explored, but it is generally thought to promote tumor growth [53].

The secretion of SASP alters the proliferation of CD3+ T cells by immune modulation and senescence reinforcement. Senescent cells can promote the senescent phenotype in nearby cells, including immunological cells, by releasing SASP factors [52]. The functioning of CD3+ T cells and their ability to mount an immunological response could be impacted by this reinforcement of senescence. By affecting the functionality and activation of immune cells, particularly CD3+ T cells, SASP factors can modify immunological responses [52].

### The Molecular Processes Responsible for Triggering SASP Expression in the Tumor Microenvironment

The types of proteins secreted by senescent cells depend heavily on the specific type of cell and the cause of senescence. For instance, even if exposed to the same level of irradiation, fibroblasts and epithelial cells produce considerably different secretomes. Furthermore, replicative senescence leads to the expression of multiple inflammatory genes in fibroblastic cells but not in endothelial or epithelial cells [54]. The transcription factors NF-kappa-light-chain-enhancer of activated B cells (NF-kB) and CCAAT/enhancer-binding protein beta (C/EBPb) play critical roles in activating SASP genes after DNA damage [55]. Additionally, the expression of C/EBPb is elevated during senescence and can be stimulated through extracellular signal-regulated kinase phosphorylation [55].

New research has revealed a further internal control process of the SASP that involves cytoplasmic DNA [56]. The studies have found that senescent cells have higher levels of cytoplasmic DNA originating from all parts of the genome, mostly from a long interspersed element-1 (LINE-1) [57]. This is the major group of repetitive elements in both human and mouse genomes.

## 5. Senotherapy for Various Cancers

According to Xu et al. (2019), the expression of amphiregulin (AREG) SASP factor by senescent stroma in human prostate cancer samples was linked to higher expression of PD-L1. Additionally, the expression of AREG by a human prostate fibroblast cell line was enough to stimulate the expression of PD-L1 on PC3 cancer cells. The PD-1/PD-L1 pathway is a significant factor in immunosuppression within the cancer microenvironment, and the overexpression of PD-L1 on cancer cells by AREG could be responsible for the considerably lower progression-free survival rates witnessed in prostate cancer samples with high levels of senescence [58].

One of the most powerful risk factors for cancer is growing older. Cell senescence, is a key barrier to tumor growth because it shields cells with dangerous mutations from spreading. Because cellular senescence can block carcinogenesis, cells must avoid oncogene-induced senescence to develop into full-blown malignancy [59]. In many kinds of cancer, the genes encoding the p53, p16, and pRb proteins, which are involved in cell senescence, are commonly altered. Senescent cancer cells appeared to be fully harmless to the body because they were non-proliferating, so therapy-induced senescence (TIS) became a necessary effect for cancer treatment [60]. The primary reason for concern regarding senescent cells is their ability to release various substances that alter the microenvironment, which can facilitate cancer development. This effect, known as SASP, involves the production of several biologically active proteins that promote carcinogenesis. However, senescent cancer cells present other risks as well. Recent research suggests that senescence may not necessarily represent the end of growth. Instead, TIS, which is a form of adaptation, may enable cancer to reoccur by avoiding the toxicity of chemotherapy and radiation. Therefore, it is essential to develop therapies that can efficiently eliminate senescent cancer cells [61,62].

Senescence plays a significant role as a destiny for NSCLC and HNSCC cancer cells that manage to survive treatment with CDDP or Taxol [35]. While senescence can curb cancer growth and enhance tissue repair in young individuals, it can pose a risk in older tissue due to the buildup of senescent cells. This is because such cells can revert and generate faulty daughter cells that may eventually transform into dysplastic cells [35]. Senolytic drug development aims to target certain cellular components such as ephrins (EFNB1 or 3), PI3KCD, p21, BCL-xL, and plasminogen-activated inhibitor-218. Among these, drugs that selectively target the BH3 domain of Bcl-2 family proteins have demonstrated senolytic activity in non-cancerous cells that have undergone replicative senescence [63]. A plethora of researchers have observed that CIS cells exhibit increased expression of Bcl-xL, which is reversed with pano-treatment and leads to cell death. Other studies have also shown that HDACi can reduce Bcl-xL expression by inhibiting transcription through histone deacetylation close to Bcl-xL transcription start sites [64]. Similar to these studies, it was also observed that CIS cells had reduced histone acetylation (measured by H3), and pano reversed this effect. While pano may also affect other genes important for senescent cell survival, further testing is necessary to confirm this hypothesis [35]. ABT263, a senolytic drug, effectively treated chemotherapy-induced senescence in cancer cells in a breast- and lung-cancer xenograft model [49,65]. When administered after treatment with DNA-damaging chemotherapeutic agents such as etoposide or doxorubicin, it led to prolonged tumor suppression in animals carrying tumors [66]. Similarly, ABT263 was successful in eliminating senescent malignant meningioma cells induced by gemcitabine treatment followed by ionizing radiation in a xenograft model [65]. Senolytic therapy given after conventional cancer treatment could therefore enhance therapeutic outcomes and effectively delay disease recurrence.

### 5.1. Treatment-Induced Cellular Senescence (TIS)

Treatment-induced cellular senescence (TIS) is a stable growth arrest state brought on by widespread cancer therapies such as chemotherapy and radiation. Therapy-induced senescence can have various effects in an oncogenic setting. It works as a tumor-suppressive mechanism by preventing cellular proliferation and by promoting immune-cell infiltration [67]. It serves as a tumor-promoting factor by promoting the growth of bystander cells and facilitating metastasis. Several pro-inflammatory cytokines and tissue-remodeling factors collectively referred to as the senescence-associated secretory phenotype (SASP) are differentially expressed and secreted, and this dual role is primarily explained by these differences [67].

A popular strategy is the so-called “one-two punch”, in which cancer cells are first rendered vulnerable with a drug that induces senescence and then destroyed with a senolytic agent [67]. An exciting area of research focuses on one-two punch cancer therapy that targets TIS. Clinical doses of cancer treatments kill tumor cells (first punch), but they also cause senescence in both tumor and normal tissues. Immune surveillance typically clears senescent cells. The expression of SASPs and senescent cell anti-apoptotic pathways (SCAPs), cellular plasticity, the tissue of origin, and cell lineage are all indicators of the heterogeneity and dynamic nature of therapy-induced senescent cells [68]. Senotherapeutic (second punch) treatment can stop tumor metastasis, recurrence, and the emergence of treatment resistance by selectively clearing senescent cells from tumors. Similar to this, selective clearance of senescent cells in healthy tissue within a dynamic spatiotemporal environment will aid in the restoration of tissue homeostasis by preventing, treating, and mitigating therapy-induced side effects [68].

### 5.2. Immuno-Senescence and Tumor Growth

Immunosenescence is the term used to describe age-related changes in immunological functions, such as lowered acquired immunity to infection, pro-inflammatory characteristics, and a higher risk of developing autoimmunity. With age, the proportions of memory-phenotype T cells in the peripheral T cell population steadily rise, but it is still unclear how this change relates to immunosenescent phenotypes [69,70]. It has been recently discovered that a genuine age-dependent T cell population known as senescence-associated T (SA-T) cells exists, which are a minor memory-phenotype CD4+ T cell subpopulation that expresses PD-1 and CD153 constitutively. The proliferation of T cells mediated by T cell receptors and the production of T cell cytokines are both defective in SA-T cells, which are characterized by cellular senescence. However, SA-T cells secrete many unusual pro-inflammatory cytokines, such as osteopontin and chemokines, in response to the stimulation of T cell receptors, resembling the SA-secretory phenotype. Further, SA-T cells accumulate and cause persistent inflammation in tissues after a variety of insults, which involves immune complex deposition, metabolic stresses, vascular damage, and tumors, alongside ageing [71]. The characteristics of leukemia-associated CD4+ T cells are similar to those of SA-T cells found in normal aged mice, encompassing senescence characteristics [72], indicating that systemic leukemia allows a swift increase in CD4+ T cell senescence. The aggressive inflammation of cancer tissues has long been known to predispose to cancer progression. As a result, an increase in SA-T cells with potent inflammatogenic function in tumor tissues could have an enormous effect on cancer progression. Cancer risk increases with age, and the role of immunosenescence in this process has been a source of concern. In particular, a recent study found that genetically eliminating tissue-resident senescent cells substantially boosts the lifespan of mice, who also have lower cancer mortality rates [73]. Hence, T cell senescence may play a role in cancer growth and advancement.

## 6. Meta-Analysis of Chemotherapy and Chemotherapy-Senolytic Combination Therapy in Cancer Patients

### 6.1. Methods: Study Design, Search Strategy, and Study Selection

Our study adhered to the preferred reporting items for systematic reviews and meta-analysis statements. To identify relevant studies, a relevant literature search was conducted on PubMed (up to 2020). Without using any automatic filters, the following keywords and their combinations were used: “Navitoclax or ABT 263 or BCL-2 inhibitor,” etc. The references of the chosen papers were further checked for any more pertinent studies. Data from abstracts and data that had been designated as unpublished were not used. Discussions among the researchers were used to settle disagreements regarding which studies should be included.

#### 6.1.1. Inclusion and Exclusion Criteria

Studies using a historical control or cohort design, cross-sectional studies, case-control studies, and research using pre-existing databases were considered suitable for inclusion. Studies that reported on a group of patients who received chemotherapy alone and navitoclax in combination were desirable for inclusion. Every study that met the criteria to be included in this evaluation included detailed information on the techniques employed to determine the drug’s effectiveness. Only the most recent and comprehensive datasets from investigations that revealed comparable patient data were taken into account. Articles were excluded if no raw data were available or if data were duplicated.

#### 6.1.2. Data Extraction

A preformatted sheet with a set of predetermined parameters was used to only extract data from the original articles: Type of therapy, the number of patients, age, medicine and dose administered, period of treatment, follow-up time, and cancer type.

### 6.2. Statistical Analysis

The data were processed and analyzed using RevMan 5. A meta-analysis for determining the efficacy of combinational therapy with navitoclax was attempted for studies presenting overall response-rate results from patients who had received traditional therapy as well as a combination of senolytic therapy. The findings were presented in the form of weighted mean differences (95% confidence interval (CI)). If *p* < 0.05, the results were considered significant.

### 6.3. Results: Study Characteristics

**Figure 4:**
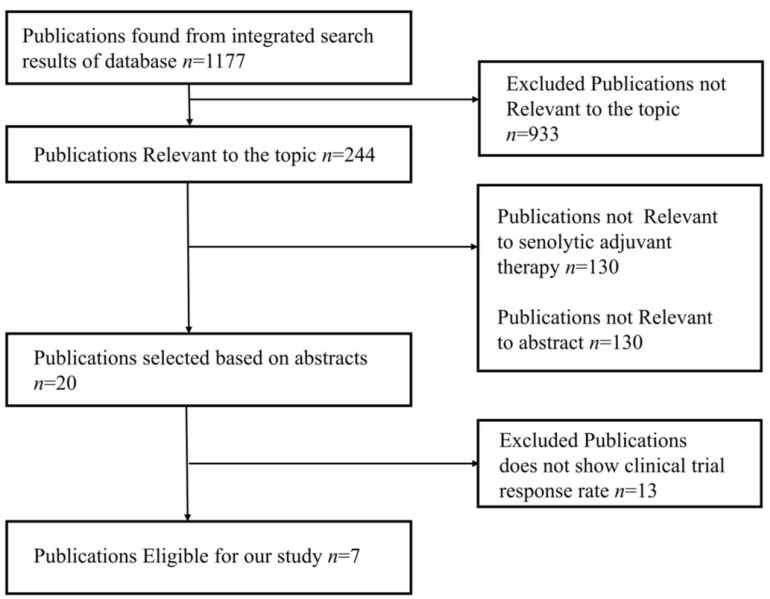
PRISMA flow diagram represents study design of meta-analysis.

The studies were chosen using the PRISMA flow diagram, which is shown below in Figure 4. For the review and meta-analysis, seven full-text articles published between 2012 and 2020 that included more than ten patients in each study were reviewed.

#### Meta-Analysis on Comparing the Efficacy of Navitoclax in Combination with Standard Conventional Chemotherapy

**Figure 5:**
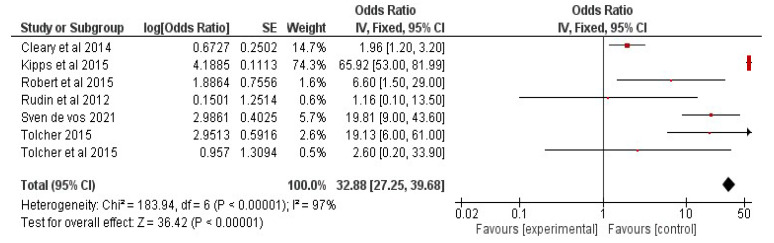
Meta-analysis result: Forest plot depicting the outcome of the study (References [74,75,76,77,78,79,80]). Red color depicts the median of the population.

A meta-analysis was performed to determine the efficacy of navitoclax in terms of overall response rate in treated patients. For the analysis, the overall response rate was calculated with 95% CI for patients who underwent monoclonal antibody therapy alone, chemotherapy, or a combination of navitoclax and senotherapy. The Q-test statistic was chosen to investigate trial heterogeneity. Additionally, the I2 value, which measures the proportion of overall variability attributable to study heterogeneity, was calculated using RevMan 5. We thank the Cochrane community for making it available as free software to conduct the forest plot analysis for this study. A study comparing the efficacy of navitoclax with other standard therapies, using the overall response rate parameter, was performed. Hence we selected seven studies for the analysis (i) Navitoclax as a single agent [74]; (ii) Navitoclax as a single agent for those who had already undergone chemotherapy [75]; (iii) Navitoclax in combination with rituximab [76]; (iv) Navitoclax in combination with gemcitabine [77]; (v) Navitoclax in combination with rituximab [78]; (vi) Navitoclax in combination with erlotinib [79]; (vii) Navitoclax in combination with irinotecan [80]. Figure 5 depicts the overall response for stand-alone therapies and combination therapies based on the findings of various studies. The pooled HR for the overall response rate with standard therapies is 32.88 (95% CI 27.25–39.68) with I^2^ = 97% and *p* = 0.00001. Furthermore, the overall response rate (ORR) for a standalone treatment with navitoclax in combination with antibody therapy in cancer patients shows the better effect, the pooled HR being 1.16 (95% CI 0.10–13.50) and the HR of patients treated with navitoclax+ chemotherapy was 2.60 (95% CI 0.20–33.90). This plot depicts that senotherapy given to patients in combination with chemotherapy or antibody therapy showed better effect when compared to stand-alone therapy.

## 7. Discussion and Conclusions

Some plant-derived senolytics such as quercetin and fisetin are sold as dietary supplements and are regarded safe when used as directed. However, given the reports of concerns with dietary supplement composition and standardization, producers should confirm the safety and efficacy of dietary supplements. Chemical modification of senolytic substances produced from plants may improve their anti-senescence efficacy. In senescent fibroblasts, for example, a curcumin [76], abn—in particular, its analogue, named EF24—demonstrated enhanced pro-apoptotic activity when compared to the mother molecule [77].

Senolytics offer the potential to enhance the effectiveness of cancer treatment while minimizing negative consequences. In cancer patients, chemotherapy or radiotherapy often led to senescence in both healthy tissue and malignant cells, with senescence-like cellular pH acidification and the restriction of healthy cell regeneration and self-repair capacity [77,78] resulting in side effects and diminished treatment effectiveness. Senescent cells have been implicated in promoting tumor relapse and resistance to therapy. These cells can contribute to the regeneration and repopulation of tumor cells after treatment, leading to disease recurrence. Targeting senescent cells through senotherapy approaches may improve the effectiveness of cancer therapies and reduce the likelihood of relapse [81]. Senescent cells can develop resistance to chemotherapy, radiation, and targeted therapies. These resistant cells can persist within tumors and contribute to treatment failure. Senotherapy has the potential to sensitize therapy-resistant cells to subsequent treatments, enhancing their effectiveness and overcoming resistance mechanisms [82]. Subsequent research should focus on using senolytics and senomorphics appropriately based on the type of cancer. In terms of senolytics, it is crucial to examine their long-term effects on tissue turnover. The elimination of senescent cells may potentially increase the workload on the remaining cells, which could ultimately lead to tissue dysfunction. The development of efficient cancer treatments would benefit from further investigation of the effects of senolytics and senomorphics, in conjunction with a better understanding of context-dependent SASP actions in vivo. Continued research and clinical trials will further elucidate the full potential of senotherapy and its integration into standard cancer treatment protocols, ultimately improving patient outcomes and quality of life [81]. The field of senotherapy is rapidly evolving, with an increasing number of preclinical and clinical trials. These studies are essential for evaluating the safety and efficacy of various senolytic interventions in humans and further advancing the field toward practical applications.

The future prospects and challenges in the field of senotherapy, including the optimization of senotherapeutic interventions, identification of biomarkers of senescence, and development of personalized treatment strategies is necessary. It is worth noting that while senotherapy holds great promise, there are still significant challenges to overcome. These include identifying optimal targets, minimizing off-target effects, ensuring long-term safety, and developing cost-effective and scalable therapeutic approaches. Nevertheless, with continued research and technological advancements, senotherapy has the potential to revolutionize the way we approach aging-related diseases and extend healthy human lifespan. Additionally, the potential implications of senotherapy in cancer prevention and longevity should be contemplated.

## Data Availability

Not applicable.
